# Grassland forage legumes and drought mitigation – sheep wool application improved soil water availability and enhanced drought–stress responses

**DOI:** 10.1002/jsfa.70841

**Published:** 2026-07-08

**Authors:** Siân Bethan MacKintosh, Rhyn Fychan, John Walter Davies, Huw Gareth Powell, Mark Boileau Scott, Christina Louise Marley

**Affiliations:** ^1^ Institute of Biological, Environmental and Rural Sciences (IBERS) Aberystwyth University Gogerddan UK

**Keywords:** water activity, red clover, lucerne, alfalfa, climate change adaptation

## Abstract

**Background:**

Sheep wool may reduce drought stress in grasslands when used as a soil amendment. Two experiments were conducted to explore if: (i) water absorbed by wool would be biologically ‘active’ and therefore available to plants, using a novel method of measuring water activity (*a*_w_); and, (ii) adding wool to compost would delay water stress in forage legumes and support plant growth. Experiment 1 determined *a*_w_ of wool pellets and potting mixtures of 0%, 7%, 14% *w/w* wool in compost (no plants). Experiment 2 parts a and b used these potting media mixtures to assess *a*_w_ on droughted and watered red clover and lucerne over 2 weeks, respectively.

**Results:**

In Experiment 1, wool pellets dry matter content was 915 g kg^−1^ and *a*_w_ < 0.473, indicating very low *a*_w_. Addition of ≥ 30% *w/w* water to wool pellets, or mixing pellets with compost, increased *a*_w_ to > 0.900, confirming high water availability. In Experiment 2, under drought, wool incrementally delayed water stress by ≥ 5 days, compared to compost. Effects of water stress on red clover and lucerne differed, with watering regime having greater influence than wool addition. The 14% wool treatment improved leaf water content of red clover. Wool addition reduced lucerne root weight, length and surface area (*P <* 0.05).

**Conclusion:**

Wool increased water retention in potting media, and the water was chemically available. Wool addition delayed drought–stress responses for red clover and altered the roots of lucerne. Further studies are needed under field conditions to understand the potential of wool on drought mitigation. © 2026 The Author(s). *Journal of the Science of Food and Agriculture* published by John Wiley & Sons Ltd on behalf of Society of Chemical Industry.

## INTRODUCTION

Agriculture is facing an urgent need to increase its sustainability^1^ whilst also reducing environmental harms and simultaneously contending with climate change. The sustainable management and efficient use of natural resources is one key strategy to achieve this.^2^ To this effect, this article considers the use of raw kemp sheep wool as potential soil amendment to support the growth of red clover (*Trifolium pratense*) and lucerne (*Medicago sativa*) under imposed drought. These legumes were selected due to their potential to contribute to a net zero approach to efficient grazed livestock production. The legumes are protein‐rich, providing an excellent feed source for grazing livestock, increasing productivity and nutrient‐use efficiency.^3^ Climate change is expected to increase both the severity and incidence of droughts, with significant effects on the productivity of agricultural land,^4^ potentially affecting the productivity of forages, such as red clover and lucerne.^5^, ^6^

Sheep wool is a natural, biodegradable, renewable fibre produced annually as a by‐product of sheep production. The International Wool Textile Organisation figures state that each sheep produces on average approximately 4.5 kg of wool per year. The global sheep population in 2022 was 1.296 billion,^7^ wool is therefore a substantial resource. Coarse wool (> 32.5 μm fineness), typically associated with hill and mountain breeds,^8^ has low economic value. Coarse wool contains medullated fibres known as kemp. Kemp fibres are brittle, cannot absorb dye, and of limited use to the textile industry.^9^, ^10^ Sheep wool value has declined over recent decades, to the extent that the cost of shearing some breeds is greater than the financial returns for the wool. Yet, sheep wool is a versatile fibre,^11^ and valorisation of kemp wool would utilise a key natural resource.^9^

Wool has been used as an economically feasible fertiliser for plant growth, due to the presence of nitrogen and sulphur that becomes available to plants as it decomposes.^12^ Additionally, sheep wool used as a soil amendment can increase soil moisture retention and increase microbial biomass.^13^, ^14^, ^15^ However, the percentage of root colonised by vesicular arbuscular mycorrhiza was plant‐specific, where basil (*Ocimum basilicum*) plants were unaffected, but thorn apple (*Datura innoxia*) had a reduction in mycorrhiza in response to wool.^15^ Where wool was used as a mulch, it improved fruit productivity and quality of plum trees grown under drought,^16^ and also reduced fluctuations in soil temperature and improved soil biological activity.^17^

Whilst information regarding dynamics of wool–water interactions is extensive for the textile industry, there is comparatively limited information regarding using wool as a soil amendment. Sheep wool has a hydrophobic cuticle, formed of scales, giving wool the necessary water‐proofing properties required by sheep. However it is a hydrophilic protein fibre with a hygroscopic core, that is able to absorb up to 30% of its weight in water vapour.^10^, ^18^ This article sought to explore whether wool could retain more moisture in the potting medium than compost alone and, using a novel approach, considered whether this moisture was chemically available to plants during imposed drought. Soil‐moisture and plant interactions are complex, as previously reviewed.^19^ One repeatable, fast method to measure the water potential of soil utilises a water activity (*a*_w_) meter to assess very dry soils.^20^ However, to the authors' knowledge, *a*_w_ has not been used to assess the effects of soil amendments on water potential. In fact, *a*_w_ is used to predict microbial spoilage potential of foods by assessing the chemical availability of water in a substrate.^21^ Importantly, *a*_w_ is distinct from moisture readings, as a medium may have high moisture content, yet low *a*_w_, indicating that water content is high, yet chemically unavailable. Thus, *a*_w_ considers the difference between free *versus* chemically‐bound water.^21^ The *a*_w_, defined as ‘the ratio of partial pressure of water vapour in a product to that of pure water’ is measured on a scale of 0.000–1.000, where 1.000 *a*_w_ is the value of pure water, and 0.000 is an anhydrous substance.^22^ An *a*_w_ of 0.600 is a common lower limit for the three domains of life, Archaea, Bacteria and Eukarya, with most bacteria requiring an *a*_w_ of > 0.900.^23^, ^24^ It has been hypothesised that sheep wool may resist degradation by bacteria and fungi due to hygroscopic nature of wool fibres, preventing the use of absorbed water by microbes to facilitate degradation.^25^ Here, by testing the effect of wool on potting media *a*_w_, we aimed to demonstrate whether water absorbed by wool is chemically available, which would be indicated by an *a*_w_ > 0.900. Water potential of low moisture soils has been previously explored using *a*_w_,^20^ however the authors believe this is the first time *a*_w_ has been used to study the impact of wool used as a soil amendment.

The following hypotheses were tested in this study, (i) that wool would increase the water held in the potting media, and this water would be chemically available to plants; (ii) that addition of wool to the potting media mitigates the effects of drought on red clover and lucerne, through improved water potential of the potting media; (iii) that wool will enhance plant growth, independent of the watering status of plants.

Two experiments were conducted to explore these hypotheses. Experiment 1 explored the effect of wool on the hydrological processes of the growth medium in the absence of plants. Experiment 2 parts a and b considered the plant response to the different potting media mixtures under droughted and watered conditions, for two forages, red clover and lucerne, respectively.

## MATERIALS AND METHODS

### Preparation of potting media

Wool pellets (1 cm × 0.5 cm pellet size) of raw Dolgellau type kemp wool (British wool grade 766) through a pelletising mill (Ecokraft AG, Sanierung, Germany). Sustainable Innovations (Asheridge, UK) and commercial compost (John Innes No. 3, Westland) was used in all potting mixtures.

Wool pellets and compost were mixed on a dry matter (DM) basis to achieve three potting media mixtures: (1) 0% wool (0:100 *w/w* wool/compost mixture), (2) 7% wool (7:93 *w/w* wool/compost mixture), (3) 14% wool (14:86 *w/w* wool/compost mixture). The oven DM content of wool pellets, compost and potting mixtures were assessed after drying at 100 °C for 48 h.

### Experiment 1. Hydrological behaviour of wool pellets

To assess the availability of water absorbed by the wool pellets, *a*_w_ was assessed for wool pellets unwetted (ambient environment) and pellets soaked for 10 min in 10%, 30%, 50% or 70% (*w/w*) distilled water. Potting media mixtures (0% wool, 7% wool and 14% wool) were also tested. The *a*_w_ measurements were measured in duplicate using an *a*_w_ meter (Lab Master AW neo, Novatron Scientific, East Grinstead, UK; 24 °C chamber temperature), reported to three decimal places.

To establish the effect of wool on the moisture content relative to pot capacity of each potting mixtures (0% wool, 7% wool and 14% wool), each mixture was prepared in triplicate without plants. Pot capacity refers to the relative amount of water present in the potting mixtures, measured gravimetrically, as a percentage of the maximum water‐holding capacity of that medium.^26^, ^27^ The potting mixtures were fully saturated with water to establish 100% ‘pot capacity’ by repeatedly wetting from above and keeping the pots in standing water for 1 h. Pots were then drained for 2 h before re‐weighing to calculate the soil water content (SWC) at 100% pot capacity by:

$$\begin{array}{l} \mathrm{SWC}\;\mathrm{at}\;100\%\mathrm{pot}\;\text{capacity}=\mathrm{Wet}\;\mathrm{pot}\;\text{mass}\\ \;-\;\mathrm{DM}\;\text{mass of potting media}-\text{Empty}\;\mathrm{pot}\;\text{mass} \end{array}$$

The corresponding moisture content of the fully saturated growth medium (equivalent to 100% pot capacity) was measured using a HH2 moisture meter fitted with a SM200 probe (Delta Devices, Cambridge, UK). Following saturation, the potting mixtures were placed in a controlled environment chamber at 20 °C/12 °C day/night temperature, with a 10 h photoperiod (light intensity 350 μmol m^−2^ s^−1^), 70% humidity for 32 days, to assess evaporative losses, in the absence of plants. No additional water was applied after initial saturation. Every 2–3 days, whole pot weight and average of three moisture readings were evaluated and recorded to calculate the percentage of pot capacity using the following equations:

$$\mathrm{SWC}\left(g\right)=\begin{array}{l} \text{Whole}\;\mathrm{pot}\;\text{mass}-\text{Container mass}\\ -\;\text{Potting media}\;\mathrm{DM}\;\text{mass} \end{array}$$

$$\mathrm{Pot}\;\text{capacity}\;\left(\%\right)=\left(\mathrm{SWC}÷\mathrm{SWC}\;\mathrm{at}\;100\%\mathrm{pot}\;\text{capacity}\right)\times 100$$

### Experiment 2. Effect of wool on plant growth

#### Experimental design and treatments

Red clover (cv. AberClaret) and *Rhizobium melitoti*‐inoculated lucerne (var. Timbale) plants were set up as separate experiments (Experiment 2a and Experiment 2b, respectively) and the same experimental design and methodology used for each forage. Three potting mixtures were tested (0% wool, 7% wool and 14% wool), along with two water treatment groups, ‘watered’ or ‘droughted’; yielding a three (wool inclusion) by two (watering regime) design with *n* = five plant replicates per treatment. A randomised block layout, with one replicate per block (plant location), and a total of five blocks was used for plant replicate layout. The experimental blocks were surrounded by guard plants of the corresponding plant species to reduce edge effects.

**Figure 1:**
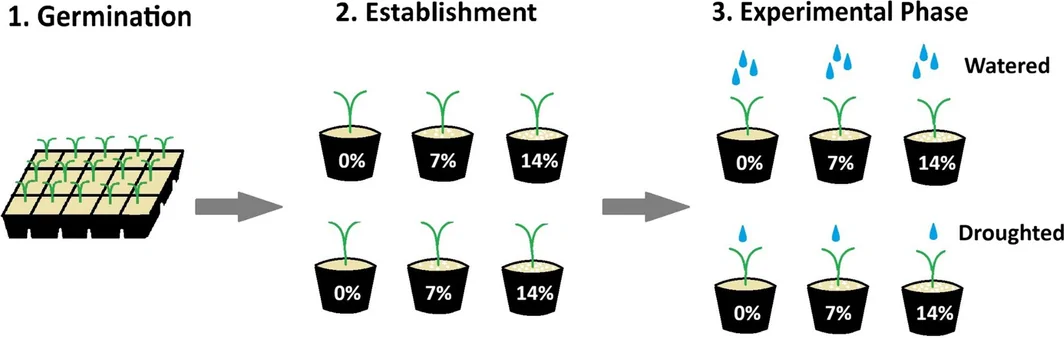
Experiment 2a and Experiment 2b set‐up of the red clover and lucerne plants, respectively. In step 1, seeds were sown in compost alone and grown on for 2 months. Step 2, plug plants were transferred to individual pots containing the 0% wool, 7% wool or 14% wool potting media mixtures and established for 1 month. Step 3 (experimental phase) where plants were maintained either well watered (55 mL per pot, daily) or exposed to drought conditions with restricted watering (20 mL per pot at 2‐day intervals) over a 2 week‐period.

Plant growing procedure is summarised in Fig. 1. Seeds were sown in compost alone in seed‐plug trays (4 cm diameter by 5 cm depth) and established in a glasshouse. After germination, each plug was thinned to one plant per plug and established for 2 months, then pruned to a fixed height (7.5 cm) and transplanted to 15 cm wide by 14 cm deep plant pots (one plant per pot) containing the respective potting mixtures (0% wool, 7% wool or 14% wool, *n* = ten plants per mixture). Plants were then maintained for 1‐month in the potting mixtures in a controlled environment facility (20 °C/12 °C day/night temperature, 70% humidity, 10 h photoperiod, light intensity 350 μmol m^−2^ s^−1^) before the experimental drought period was implemented.

During the establishment period, all pots were assessed daily for potting medium moisture using HH2 moisture meter fitted with a SM200 probe (Delta Devices) before being watered. During the 2‐week experimental period, ‘watered’ treatment plants were watered daily with 55 mL per pot. The amount of water applied to these pots was determined during the establishment period to maintain the moisture of the potting mixtures at approximately 40%. Plants assigned to the ‘droughted’ treatments were watered once every 2 days with 20 mL per pot. Plants were harvested at the end of the experimental period.

### Measurements

#### Potting mixture assessments

Potting mixture moisture content (hereafter referred to as pot moisture) was recorded daily and at harvest as described earlier. At harvest, a 50 g cored sub‐sample of potting mixture was removed from each pot for *a*_w_ assessment. Soil cores were stored at room temperature in a sealed container until measurement using an *a*_w_ meter, as described earlier.

Previous reports identified that wool used as a soil amendment can release sodium into soil,^15^, ^28^ increasing soil salinity,^28^ potentially causing osmotic stress to plants. Therefore, the salinity of the potting mixture was measured, after herbage had been harvested, by flooding all pots with water to equalise the moisture content and allowed to drain until downwards, free moving water ceased. Electrical conductance (EC) was measured as an indicator of salinity using Field Scout probe (Spectrum Technologies Inc., Aurora, IL, USA). Pots were stored at 4 °C for subsequent root structure assessments.

#### Herbage assessments

The uppermost fully open leaf (without the petiole) was assessed for leaf turgidity, using leaf relative water content (RWC) as a proxy. Individual leaves were weighed immediately after harvest (harvest mass) to four decimal places. Each leaf was then floated in a container of distilled water (temperature ~15 °C), with the underside of the leaf in contact with the water for 4 h in darkness. Leaves were removed, gently patted dry and re‐weighed (turgid mass) before being oven dried for 24 h at 85 °C and re‐weighed (DM mass). The RWC was calculated as:

$$\mathrm{RWC}\left(\%\right)=\begin{array}{l} \left(\text{harvest mass in grams}-\mathrm{DM}\;\text{mass in grams}\right)\times 100\\ ÷\left(\text{turgid mass in grams}-\mathrm{DM}\;\text{mass in grams}\right) \end{array}$$

The next three uppermost leaves were imaged to measure leaf area using the Delta‐T WinDIAS 3 image analysis system (Delta‐T Devices, Cambridge, UK).^44^ To assess herbage yield, all remaining herbage was removed at the level of the potting mixture surface. Herbage freeze dry yield was assessed after drying at 100 °C for 48 h.

#### Root analysis

Roots were washed through a 1 mm sieve to remove compost and wool before being imaged using a flatbed scanner (Epson Perfection V850 Pro, Watford, UK) and then oven dried at 100 °C for 48 h. Root images were analysed using WinRHIZO software (Regent Instruments Inc., Quebec, Canada) to measure root architecture length and diameter. Root DM content was determined for the tap root and fine roots independently and the root‐to‐shoot ratio calculated by dividing the root DM biomass by the herbage DM biomass.

#### Water use efficiency (WUE)

Water use efficiency (WUE) was calculated as previously described.^29^ Briefly, plant evapotranspiration under varying conditions during the experimental phase was calculated using:

$$\text{Evapotranspiration}\;\left(\mathrm{kg}\right)=\left(I+\left({\mathrm{SWC}}_{1}-{\mathrm{SWC}}_{2}\right)-D-R\right)÷1000$$

where *I* is the volume of irrigation applied (in millilitres); SWC_1_ is the starting soil water content (in grams); SWC_2_ is the final soil water content (in grams); *D* is the soil drainage; *R* is the runoff; however, as this was a pot‐based study it is considered that there is no runoff/drainage, so these last two variables were ignored.^29^

WUE was calculated:

$$\mathrm{WUE}\left(g\;{\mathrm{kg}}^{-1}\right)=\begin{array}{l} \text{Biomass above the ground(in grams)}\\ ÷\text{Evapotranspiration(in kilograms)} \end{array}$$

### Statistical analysis

Data were analysed using Genstat software (22nd edition; VSN International Ltd, Hemel Hempstead, UK), except for repeated measures analysis of variance (ANOVA) which was conducted using SPSS (version 29; IBM SPSS Statistics for Windows, Armonk, NY, USA). Significance was attributed where *P <* 0.05.

The *a*_w_ and the SWC of potting mixtures were analysed using ANOVA, with Bonferroni *post hoc* test. The rate of decline in pot moisture following initial saturation was analysed using repeated measures ANOVA. Time points selected for analysis included the initial pot moisture and weekly measurements. Wool content of potting mixtures was included as an independent variable.

Simple linear regression analysis determined the relationships between pot capacity (independent variable) on pot moisture (dependent variable) for each potting mixture (grouping factor). Moisture equivalent of pot capacity associated with inducing severe water stress for lucerne and red clover, was then determined where pot capacity of 40% or 45% was reported to induce water stress in red clover^30^ and in lucerne,^26^ respectively.

Harvest measurements taken at harvest were analysed using ANOVA, where wool content of potting mixture and watering treatment were analysed as fixed effects with interaction, by block. Where *P <* 0.05 for main effects of the ANOVA, Bonferroni *post hoc* analysis was further conducted, unless stated otherwise. Residuals were plotted to check the normality and homoscedasticity of the data, in accordance of satisfying ANOVA assumptions. Where stated, data was transformed using log^10^ transformation to satisfy the condition of normality, and the geometric mean is expressed.

The relationship between root architecture and specific leaf area on WUE was analysed using multi linear regression analysis grouped by watering regime or wool inclusion rate. The independent variables were checked to ensure that there was no correlation, and therefore the assumption of no collinearity was met.

## RESULTS

### Experiment 1. Hydrological status of potting mixture in the absence of plants

**Figure 2:**
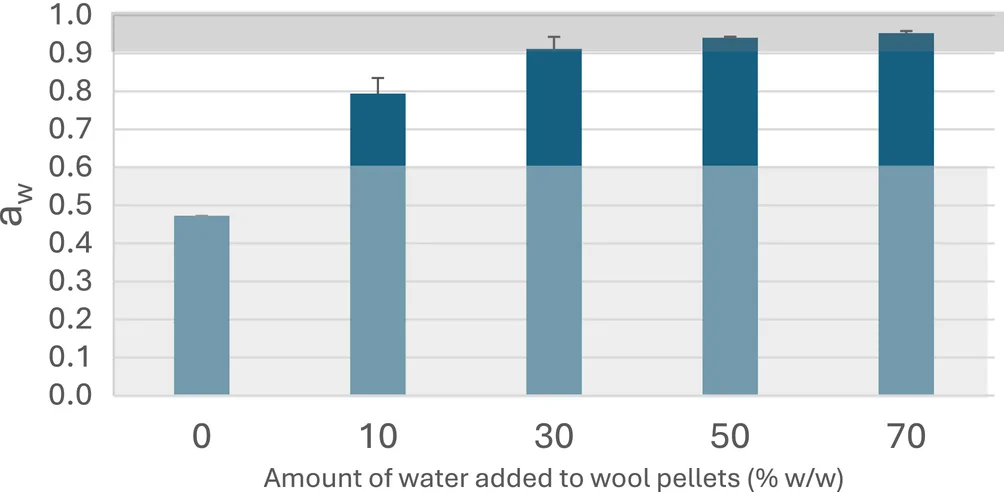
The water activity (*a*_w_) of wool pellets alone. The *a*_w_ was tested using dry wool pellets or after wetting with water (*w/w*, fresh matter basis). Most bacteria grow at an *a*_w_ > 0.900 (dark grey, upper hatched area), indicating chemical availability of water. Whereas only xerophilic moulds and osmophilic yeasts can grow at *a*_w_ < 0.600, indicating that freely available water is limited (light grey, lower hatched area).^23^ Data show the mean ± standard deviation.

Wool pellets and compost had a DM content of 899 and 634 g DM kg^−1^ fresh weight, respectively. Wool pellets alone had an *a*_w_ of 0.4726 (Fig. 2). Addition of 10% *w/w* water to wool pellets increased the *a*_w_ to 0.7937. However, addition of 30%, 50% or 70% *w/w* water to the pellets resulted in an *a*_w_ > 0.900. Once wool pellets were mixed with compost to create the experimental potting mixtures, *a*_w_ was > 0.9900 for all mixtures prior to wetting, and *a*_w_ did not differ significantly between mixtures. Compost, prior to mixing or wetting, had an *a*_w_ of 0.9941.

**Figure 3:**
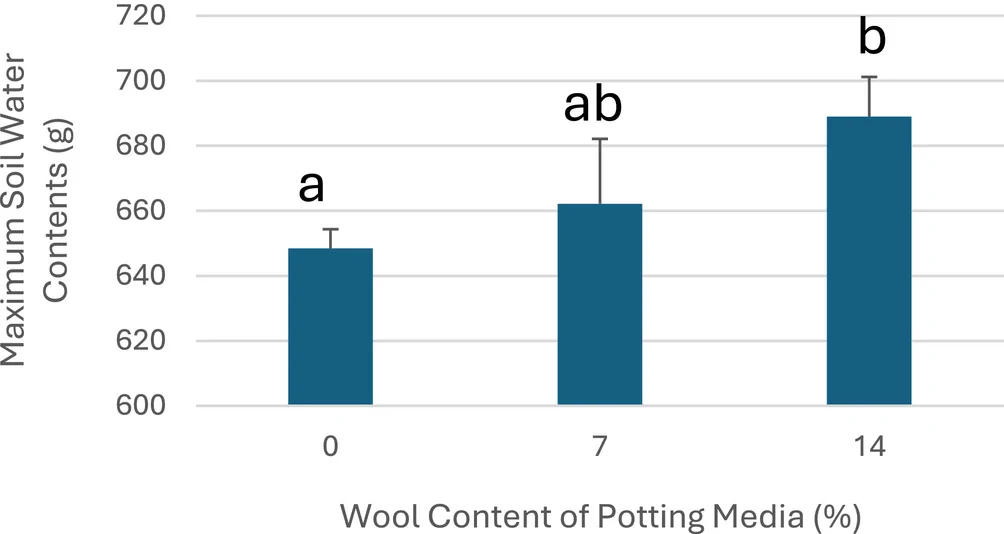
Mean maximum soil water content (SWC) of the different potting media mixtures (wool + compost) when saturated with water (100% of pot capacity). Columns with different superscript are statistically different, *P* < 0.05. Error bars indicate standard deviation.

At maximum pot capacity, the maximum SWC increased as the amount of wool increased in the potting mixture (*P <* 0.05, Fig. 3). There was a significant increase in SWC for 14% wool mixture *versus* the control (0% wool mixture) (*P <* 0.05), with the 7% wool mixture being intermediate. Following initial saturation, pot moisture declined over time when pots were stored at 20 °C/12 °C day/night for 32 days (*P <* 0.001, Supporting Information Fig. S1). Whilst wool had no significant main effect on pot moisture, there was a significant time × wool interaction on pot moisture, where compost alone had the highest moisture at the start, but lowest moisture after 32 days, relative to potting mixtures containing wool pellets (*P <* 0.05).

There was a positive, linear relationship between pot capacity and pot moisture readings using a soil moisture probe for each potting mixture (*P <* 0.001, Supporting Information Table S1 and Fig. S2). The adjusted coefficient of determination (adjusted *R* ^2^) was 96.9% with a standard error of 4.47. The simple linear regression of pot moisture against pot capacity for the 7% wool and 14% wool potting mixtures were significantly different compared to that of 0% wool (*P <* 0.05).

### Experiment 2. Effect of wool mitigating the impact of drought on plant growth

**Figure 4:**
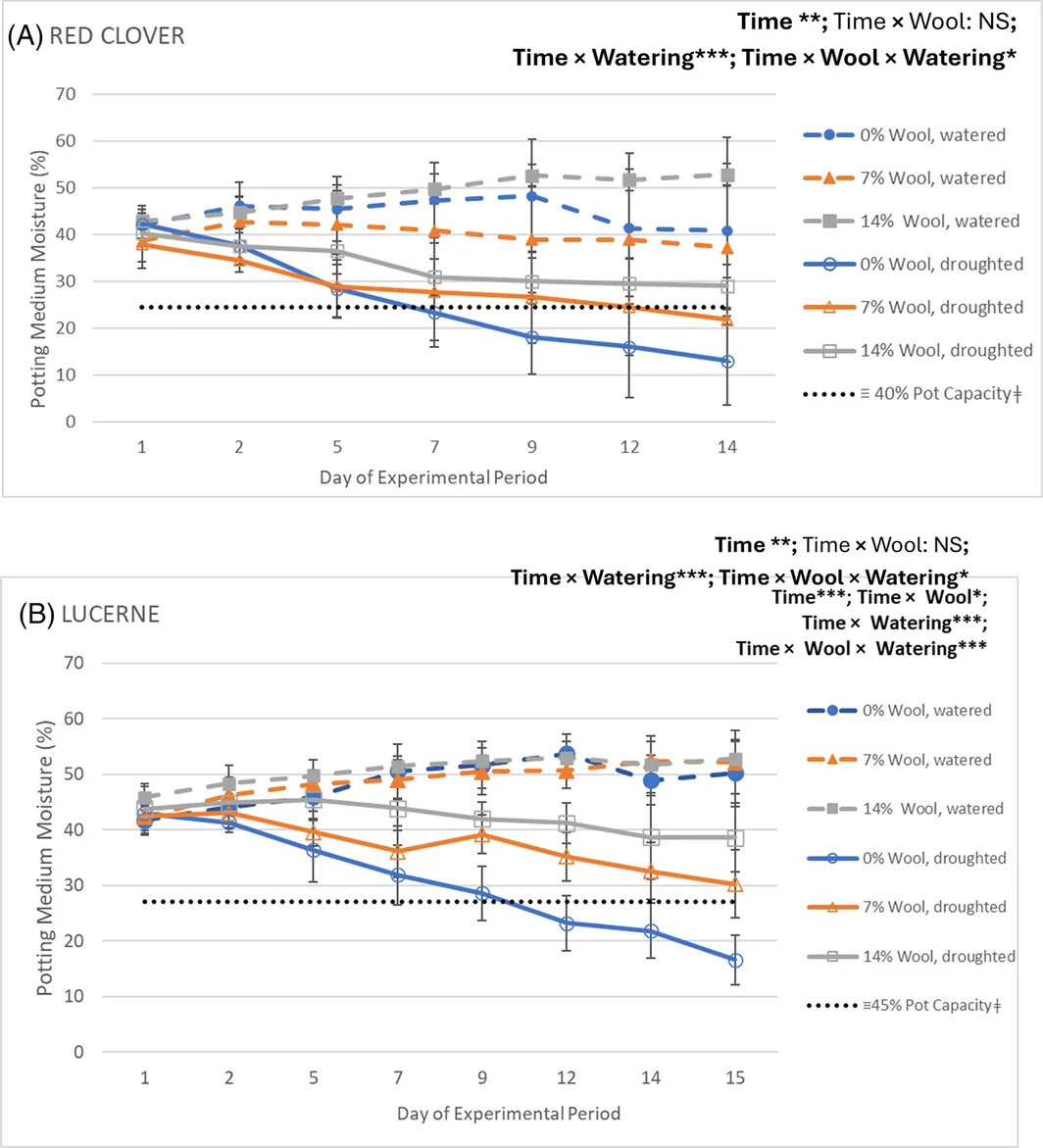
Moisture content of the potting media mixtures during the experimental period for pots planted with (A) red clover or (B) lucerne. ^ǂ^The respective average moisture threshold for reaching severe water stress for red clover and lucerne is indicated by a dashed line. The red clover 40% pot capacity equates to a pot moisture of 23.43%, 24.68% and 25.53% (average = 24.5%) for 0% wool, 7% wool and 14% wool potting media mixtures, respectively. The lucerne threshold for water stress of a 45% pot capacity, was equivalent to 26.4%, 27.3% and 27.7% (average = 27.1%) pot moisture for 0% wool, 7% wool and 14% wool potting mixtures, respectively. *P < 0.05, **P < 0.01, ***P < 0.001, NS = not significant. Data show the mean ± standard deviation.

A pot capacity of 40% has been reported to induce water stress in red clover,^30^ whereas a pot capacity of 45% has been reported to induce water stress in lucerne.^26^ Using the regression data (Table S1), the predicted pot moisture to yield a 40% pot capacity equated to a pot moisture of 23.43%, 24.68% and 25.53% (average = 24.5%) for 0% wool, 7% wool and 14% wool potting mixtures, respectively. For a 45% pot capacity, pot moisture was equivalent to 26.4%, 27.27% and 27.74% (average = 27.1%) for 0% wool, 7% wool and 14% wool potting mixtures, respectively. For graphical ease, a single line is shown on Fig. 4 representing an average pot moisture of 24.5% or 27.1% pot moisture for a pot capacity of either 40% or 45%, for red clover and lucerne, respectively.

During the experimental period, there was a significant interaction of time‐by‐watering, but also time‐by‐wool‐by‐watering observed for the red clover plants on pot moisture (*P <* 0.05, Fig. 4(A)). From day 2 onwards, droughted red clover plants had lower pot moisture than watered red clover plants, regardless of wool content (*P <* 0.001). Droughted red clover grown in 0% wool or 7% wool, reached a pot moisture that would be expected to induce water stress on days 7 and 12, respectively. In contrast, the 14% wool droughted red clover plants and the well‐watered red clover plants never reached this threshold within the experimental period.

At harvest, droughted red clover plants had a lower pot moisture than watered red clover plants (*P <* 0.05, Table 1). Addition of 14% wool resulted in a pot moisture that was higher than compost alone (main effect: wool inclusion, *P <* 0.05), with 7% wool treatment being intermediate. The pot moisture of the 14% wool treatment was 40.9%, which was numerically similar to that of predicted mean for the watered treatments of 43.7%. The salinity of the potting mixtures was affected by watering regime and wool content (*P <* 0.05), and a significant interaction of watering regime‐by‐wool was observed for red clover (*P <* 0.05). For red clover, 0% wool had the lowest salinity, regardless of watering regime compared to all other treatments (*P <* 0.05). Addition of wool increased salinity (*P <* 0.05), and this effect was incremental for watered‐red clover (*P <* 0.05). The *a*_w_ at harvest for all treatments was > 0.9. Droughting reduced the *a*_w_ of the potting mixture at harvest for the red clover plants (*P <* 0.05). Addition of 14% wool lowered potting mixture *a*_w_ at harvest compared to 0% wool, with the 7% wool group having intermediate *a*_w_ (*P <* 0.05). There was no significant interaction between watering regime and wool content.

**Table 1:** Potting media characteristics at harvest for red clover (Experiment 2a)

*Potting medium moisture (%)*					
**Main effect watering**	**Watered**	**Droughted**		**SED**	**Significance**
	43.7	21.3		4.13	***
**Main effect wool (%)**	**0%**	**7%**	**14%**		
	27^a^	29.6^ab^	40.9^b^	5.06	*

*Salinity (mS cm* ^*−1*^ *)*					
**Main effect watering**	**Watered**	**Droughted**		**SED**	**Significance**
	4.7	6.5		0.26	***
**Main effect wool (%)**	**0%**	**7%**	**14%**		
	1.7^a^	6.2^b^	9^c^	0.32	***
**Watering × Wool**	**0%**	**7%**	**14%**		
**Watered**	1.0^a^	4.5^b^	8.8^c^	0.45	***
**Droughted**	2.4^a^	7.9^c^	9.3^c^		

*Water activity (a*_*w*_ *)*					
**Main effect watering**	**Watered**	**Droughted**		**SED**	**Significance**
	0.9875	0.9805		0.00144	***
**Main effect wool (%)**	**0%**	**7%**	**14%**		
	0.9878	0.9839	0.9803	0.00177	**

*Note*: Data show the predicted means and SED. Watering by wool interaction term was only significant for salinity. Where a comparison had >2 treatment groups, means marked with different superscript indicate a significant difference within that comparison, *P > 0.05, **P < 0.01, ***P < 0.001, NS = not significant.

The pot moisture of lucerne plants during the experimental period showed a significant time × wool × water interaction (*P <* 0.05, Fig. 4(B)). Potting mixture moisture decreased over time for droughted plants, with 0% droughted lucerne plants having the lowest pot moisture by the end of the experimental period. Indeed, the 0% wool droughted plants were the only lucerne treatment group to reach a pot moisture that would be expected to induce severe water stress in lucerne plants. Addition of wool to droughted plants appeared to slow down the rate of moisture loss from the potting mixture, in an incremental manner. Watered lucerne plants had similar potting mixture moisture throughout the experimental period, irrespective of the amount of wool added.

At harvest, a significant watering‐by‐wool interaction was observed for lucerne (*P <* 0.05, Table 2). At harvest, watered plants had similar pot moisture, regardless of the amount of wool present (*P* > 0.05). Droughted plants had lower potting mixture moisture than watered plants, irrespective of wool content (*P <* 0.05), however addition of 7% wool or 14% wool increased pot moisture compared to 0% wool (droughted) (*P <* 0.05).

**Table 2:** Potting media characteristics at harvest for lucerne pots (Experiment 2b)

*Potting medium moisture (%)*					
**Main effect watering**	**Watered**	**Droughted**		**SED**	**Significance**
	51.73	28.5		1.333	***
**Main effect wool (%)**	**0%**	**7%**	**14%**		
	33.42^a^	41.21^b^	45.71^c^	1.632	***
**Watering × Wool**	**0%**	**7%**	**14%**		
**Watered**	50.24^d^	52.14^d^	52.8^d^	2.308	***
**Droughted**	16.6^a^	30.28^b^	38.62^c^		

*Salinity (mS cm* ^*−1*^ *)*					
**Main effect watering**	**Watered**	**Droughted**		**SED**	**Significance**
	4.2	5.6		0.29	***
**Main effect wool (%)**	**0%**	**7%**	**14%**		
	1.6^a^	5.4^b^	7.6^c^	0.36	***
**Watering × Wool**	**0%**	**7%**	**14%**		
**Watered**	1.6^a^	4.3^b^	6.5^c^	0.51	***
**Droughted**	1.7^s^	6.5^c^	8.7^d^		

*Water activity (a*_*w*_ *)*					
**Main effect watering**	**Watered**	**Droughted**		**SED**	**Significance**
	0.9861	0.9856		0.00137	NS
**Main effect wool (%)**	**0%**	**7%**	**14%**		
	0.9887	0.9847	0.9842	0.00168	*

*Note*: Data show the predicted means and SED. Watering by wool interaction term was only significant for salinity. Where a comparison had >2 treatment groups, means marked with different superscript indicate a significant difference within that comparison, *P > 0.05, **P < 0.01, ***P < 0.001, NS = not significant.

A significant interaction of watering regime‐by‐wool was observed for the salinity of potting mixture of lucerne plants at harvest (*P <* 0.05). Drought or addition of wool increased salinity (*P <* 0.05), and the wool effect was incremental for lucerne (*P <* 0.05). Watering regime did not affect *a*_w_ of lucerne plants (*P* = 0.69). Addition of 14% wool lowered potting mixture *a*_w_ at harvest compared to 0% wool, with the 7% wool group having intermediate *a*_w_ (*P <* 0.05). There was no significant interaction between watering regime and wool content for either plant species. The *a*_w_ at harvest for all treatments remained > 0.9.

No significant watering‐by‐wool interactions were observed for any herbage or root characteristics at harvest, for either plant species. Drought reduced plant height, lowered RWC and increased WUE in red clover plants at harvest, compared to watered plants (*P <* 0.05, Table 3). Addition of 7% wool also lowered RWC of red clover plants, compared to the 0% wool (*P* = 0.031). However, red clover grown in 14% wool had intermediate RWC. Addition of wool increased WUE for red clover plants (*P <* 0.05).

**Table 3:** Red clover plant characteristics at harvest showing the predicted means for the main effects of watering regime or wool content

	Watering regime				Wool content				
	Watered	Droughted	SD	Significance	0%	7%	14%	SD	Significance
*Herbage*									
Plant height (cm)	41.6	33.9	3.39	*	36.9	40.2	36.2	4.15	NS
DM yield (g per plant)	2.7	2.6	0.40	NS	2.3	3.0	2.6	0.49	NS
Specific leaf area (cm^2^)	11.1	8.8	1.21	NS	10.8	9.9	9.2	1.48	NS
RWC of uppermost leaf (%)	80.4	69.7	2.14	***	78.3^a^	70.9^b^	76.0^ab^	2.62	*
WUE (g kg^−1^)	4.1	7.2	0.61	***	4.04^a^	6.15^b^	6.70^b^	0.75	**
*Roots*									
Total root weight (g DM)	0.47	0.45	0.079	NS	0.44	0.53	0.41	0.097	NS
Tap root weight (g DM)	0.08	0.09	0.012	NS	0.09	0.10	0.08	0.015	NS
Fine roots weight (g DM)	0.39	0.35	0.075	NS	0.35	0.43	0.33	0.091	NS
Total root length (cm)	1674	1851	154.9	NS	1716	1742	1830	189.7	NS
Average root diameter (mm)^†^	3.96	5.76	0.080	NS	4.19	5.78	4.49	0.098	NS
Number of root tips	6320	6401	926.8	NS	7149	6092	5842	1135.0	NS
Number of root forks^†^	31 502	29 335	0.061	NS	31 386	29 391	30 453	0.08	NS
Surface area (cm^2^)	296	287	35.1	NS	307	289	277	43.0	NS
Root‐to‐shoot ratio (DM basis)^†^	−0.74	−0.76	0.074	NS	−0.65	−0.78	−0.82	0.091	NS

^†^ Log_10_ data used for analysis of variance (ANOVA). The geometric mean is shown. *Note*: There were no significant interaction between the two main effects (*P* > 0.05). Where a comparison had > two treatment groups, means marked with different superscript indicate a significant difference within that comparison, *P > 0.05, **P < 0.01, ***P < 0.001, NS = not significant. DM, dry matter; RWC, relative water content; SD, standard deviation; WUE, water use efficiency.

**Figure 5:**
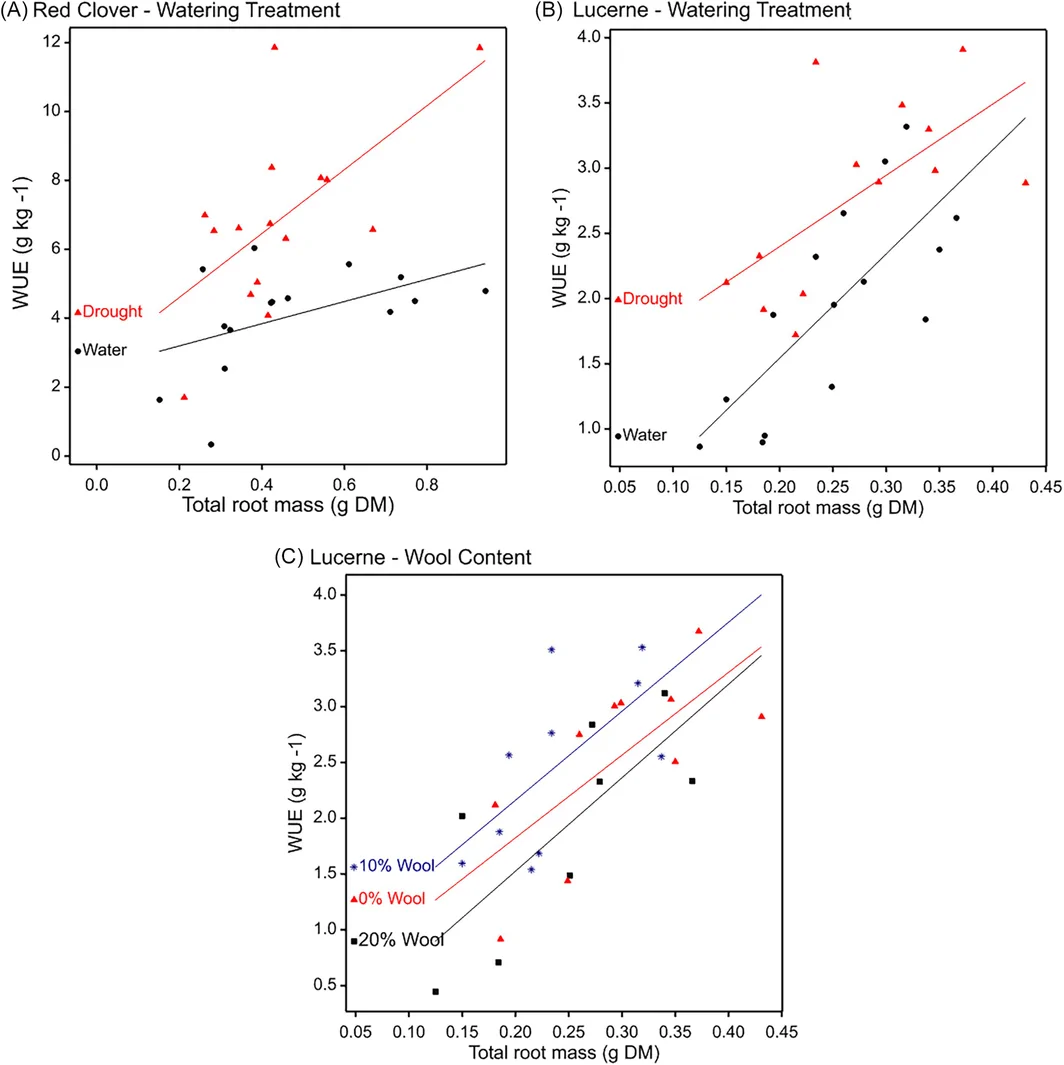
Relationship between total root weight and water use efficiency (WUE) by red clover and lucerne, grouped by either (A, B) watering regime or (C) wool inclusion.

Root architecture of red clover was not affected by either watering regime or wool content (Table 3). For red clover, the multi linear regression model of leaf area and total root mass, was significant when grouped by watering regime (*P* < 0.001, adjusted *R* ^2^ values = 48.3%, standard error 1.89, Table S2), but not when grouped by wool inclusion rate. For red clover, a significant positive regression was observed between total root weight and WUE (Table S2), where larger total root mass was associated with higher WUE (*P* = 0.006, Fig. 5).

For lucerne, drought reduced both plant height and specific leaf area of lucerne, decreased RWC and increased WUE, compared to watered plants (*P <* 0.05, Table 4). Specific leaf area was also significantly affected by wool, where 14% wool reduced specific leaf area compared to 0% wool or 7% wool (*P <* 0.05). Addition of either 7% wool or 14% wool lowered RWC of lucerne, relative to the 0% wool group (*P <* 0.05).

**Table 4:** Lucerne plant characteristics at harvest showing the predicted means for the main effects of watering regime or wool content

	Watering regime				Wool content				
	Watered	Droughted	SD	Significance	0%	7%	14%	SD	Significance
*Herbage*									
Plant height (cm)	35.1	27.5	2.59	**	33.4	30.4	30.1	3.18	NS
DM yield (g per plant)	1.3	1.0	0.15	NS	1.3	1.1	1.0	0.18	NS
Specific leaf area (cm^2^)	6.1	4.4	0.48	**	6.3^a^	5.5^a^	3.9^b^	0.59	**
RWC of uppermost leaf (%)	81.4	71.5	1.38	***	81.1^a^	74.0^b^	74.4^b^	1.69	***
WUE (g kg^−1^)	1.94	3.25	0.32	***	2.5	2.5	2.8	0.40	NS
*Roots*									
Total root weight (g DM)	0.25	0.27	0.026	NS	0.30	0.24	0.25	0.032	NS
Tap root weight (g DM)	0.08	0.09	0.009	NS	0.08	0.09	0.09	0.011	NS
Fine roots weight (g DM)	0.17	0.18	0.021	NS	0.21	0.15	0.16	0.026	NS^‡^
Total root length (cm)	1201	1149	125.1	NS	1419	1023	1083	153.2	*
Average root diameter (mm)^†^	2.75	2.51	0.056	NS	3.00	2.40	2.50	0.068	NS
Number of root tips	4424	3940	602.1	NS	5554	3443	3624	737.4	*
Number of root forks	14 878	14 371	2017.5	NS	19 592	11 363	12 919	2470.9	**
Surface area (cm^2^)	207	200	20.8	NS	247	177	186	25.5	*
Root‐to‐shoot ratio (DM basis)	0.22	0.30	0.025	**	0.24	0.25	0.27	0.031	NS

^†^ log_10_ data was used for analysis of variance (ANOVA).

^‡^ *P* = 0.05. *Note*: There were no significant interaction between the two main effects (*P* > 0.05). Where a comparison had > two treatment groups, means marked with different superscript indicate a significant difference within that comparison, *P > 0.05, **P < 0.01, ***P < 0.001, NS = not significant. DM, dry matter; RWC, relative water content; SD, standard deviation; WUE, water use efficiency.

For lucerne, watering increased root‐to‐shoot ratio (*P <* 0.05), with root architecture otherwise unaffected (Table 4). Wool addition reduced lucerne total root length, number of root tips and forks and total root surface area, compared to compost alone (*P <* 0.05). Wool addition had a tendency (*P* = 0.05) to reduce the fine root mass of lucerne plants. The 7% wool treatment had a lower total root length and root surface area than the 0% wool treatment (*P <* 0.05), with the 14% wool treatment being intermediate. Lucerne root forks and tips were lower where wool was added at either 7% or 14%, compared to 0% wool (*P <* 0.05).

The multi‐linear regression model of leaf area and total root mass on lucerne WUE, was significant when grouped by watering regime (*P <* 0.001; adjusted *R* ^2^ values = 58.9%, standard error 0.582), and when grouped by wool inclusion (*P* = 0.012; adjusted *R* ^2^ values = 42.6%, standard error 0.688) **(**Table S2). Higher root mass was associated with greater WUE (*P <* 0.05, Fig. 5).

## DISCUSSION

The hydrological status of wool pellets produced from Welsh Mountain sheep wool was explored. The potential of wool to mitigate the effects of simulated drought on red clover or lucerne plants was also examined. Wool increased the amount of water held in potting mixtures, and findings confirmed this water was chemically available, even after a period of drought. However, plant indicators of the efficacy of wool to mitigate drought, such as root architecture, RWC, plant yield and WUE were varied between forage legume species.

### Hydrological behaviour of wool pellets

The study explored whether wetted wool had low *a*_w_ values, which could potentially impact on the availability of water that is absorbed by wool to support plant growth. While others have used *a*_w_ values as a tool to measure water availability in low moisture soils,^20^ the authors believe this study is the first use of *a*_w_ to assess wool as a soil amendment.

Prior to wetting, and before mixing with compost, the wool pellets had high DM content and low *a*_w_ (< 0.6), therefore the limited moisture in the pellets was not chemically available. In agreement with previous reports that wool can absorb up to 30% of its weight in water,^9^, ^18^ *a*_w_ was only > 0.9 in wool pellets (in the absence of compost) when > 30% *w/w* water was added. When < 30% *w/w* water was added, the *a*_w_ was correspondingly lower, likely reflecting the hygroscopic nature of wool. Once wool pellets were mixed with compost to formulate the experimental potting mixtures, *a*_w_ increased to > 0.9, indicative of high water chemical availability.^24^ It appears that the relatively high moisture content of compost contributed sufficient free water to achieve high *a*_w_ in the potting mixtures. Water present in the compost appears to be absorbed by wool and then retained – as indicated by a slower decline in moisture over time. Once wetted, wool addition increased the maximum water holding capacity of the potting mixture, relative to compost alone, in a dose‐dependent manner. Others have also reported that wool inclusion in the growing media is associated with increased moisture retention.^31^, ^32^

### Potting mixture responses in the presence of plants

Droughted red clover and lucerne plants grown in compost alone resulted in a pot moisture level that was previously reported to induce water stress in these plants.^26^, ^30^ Addition of wool delayed (red clover plants), or prevented (lucerne plants), the potting mixture reaching these thresholds when exposed to drought, demonstrating a beneficial effect of using wool as a soil amendment to retain moisture within the growing medium. This effect was incrementally protective as wool inclusion increased, which may be attributed to the hygroscopic nature of wool, with its hydrophilic protein‐based fibrous structure.^10^, ^18^, ^31^, ^32^ However, the key novelty here was to test if this hygroscopically wool‐bound water was available for use by the plant. At harvest, all potting mixtures had an *a*_w_ > 0.9 (even at the lowest pot moisture after imposed drought) suggesting that the moisture held in the potting mixture was chemically available. However, addition of wool did cause an incremental reduction in *a*_w_, potentially due to increased salinity where wool was added.

In the present study, wool inclusion increased salinity in the potting mixture, up to 9 mS cm^−1^ EC, where 14% wool was incorporated into the potting mixture. Soil EC has previously been categorised as: 0–2 dS m^−1^ = not saline, 2–4 dS m^−1^ = slightly saline, 4–8 dS m^−1^ = moderately saline, and 8–16 dS m^−1^ = strongly saline.^33^ Others classify EC > 4 dS m^−1^ as saline soil.^34^ ‘Pseudo‐drought’ describes a phenomenon where there is sufficient moisture in the root zone, yet the plant is unable to utilise sufficient water to avoid water‐deficit stress.^35^ Water deficiency in plants can occur from a number of factors, including low soil moisture, but also extremes in temperature or high salinity.^35^ Salt stress can also inhibit germination and plant establishment, reduce dry matter herbage yield, reduce root length and reduce leaf water.^34^, ^36^ Red clover has been reported to have good tolerance of high soil salinity^37^; whereas other reports indicate that red clover is highly sensitive to soil salinity.^34^ In the present study, red clover DM yield, plant height and root morphology were not affected by wool content. Accordingly, based on the indicators measured in this study, there was no clear evidence of salt stress induced by wool for red clover. Lucerne has been observed to be moderately tolerant of salt stress up to 17 dS m^−1^ (equivalent to 17 mS cm^−1^), where sodium chloride is the main salt – although this was found to be cultivar specific, with some cultivars having lower tolerance.^38^ The salinity observed in the current study was well below 17 mS cm^−1^. However, as addition of wool to the potting mixture did reduce leaf area, RWC and resulted in shorter, less branched roots of lucerne plants, it is still possible that increased salinity contributed to these results. Given the increased salinity where wool was used, further monitoring the effects of wool on soil salinity and plant growth may be important.

### Red clover plant responses

Drought resulted in a reduction in plant height and RWC and an increase in WUE by red clover plants – indicating that there was a plant water stress response. Interestingly, DM yield, specific leaf area, root architecture and root‐to‐shoot ratio of red clover were not affected by either watering regime or addition of wool. For red clover plants, only the RWC and WUE were significantly affected by addition of wool. Recognised drought responses by plants include reduction in leaf water status, reduced DM yield, reduced root dry weight, increased WUE and reduced leaf area; with responses occurring in a plant‐species and drought‐severity specific manner.^29^, ^39^, ^40^ Drought is known to reduce red clover plant growth, resulting in lower plant density coverage and lower canopy height, with leaf wilting only observed after 50 days of drought.^5^ Therefore, the present study may not have imposed drought for a sufficient duration to evoke detectable changes in all the plant variables tested.

Wool addition did not improve DM yield, plant height or leaf area as hypothesised. Previous studies found wool mulch did not alter growth characteristics of plum trees (branch length, branch quality or trunk cross‐sectional area), but wool contributed to improved fruit production from the trees.^16^ In contrast, other reports found that wool used as a fertiliser or geotextile was associated with improved yield and increased plant height.^32^, ^41^ Haque and Naebe^31^ found that the effect of wool on plant height was non‐linear, with a lower inclusion of wool powder (165 mg per 150 g soil) having the greatest benefit, compared to 385 mg wool per 150 g soil – potentially due to an optimised balance in moisture retention by adding the appropriate amount of wool to the soil.^31^

RWC was used as a proxy for plant water status, where higher RWC indicates better water status.^29^ The reduction in red clover RWC by addition of wool was not in a dose‐dependent manner. Indeed, whilst red clover grown in the 7% wool treatment had significantly lower RWC compared to compost alone, the addition of 14% wool resulted in intermediate RWC.

WUE is increased in water‐deficit plants as a tolerance mechanism,^29,40^ however it is a multifactorial trait, affected by plant architecture, water availability and agronomic strategies. It is possible to strategically improve WUE,^42^ so this measure cannot be directly used as an indicator of water stress *per se*. The WUE of red clover was increased by both drought and wool addition. The regression analysis of leaf area and root mass on WUE indicated that red clover plants with greater root mass had greater WUE, with leaf area not having a significant effect on WUE. However, neither watering regime nor wool inclusion rate affected red clover root mass in the present study. The relationship between root mass and WUE was only significant when the regression model was grouped by watering regime, not wool inclusion, which given the lack of a significant effect of wool on root architecture of red clover, is as expected.

A tentative conclusion is that red clover plant water status was better maintained during imposed drought where 14% wool was added to the compost, potentially through reduced rate of decline in pot moisture and the increased WUE. A possible mechanism for wool increasing WUE could be improved nutrient provision by the wool. Martineau *et al*.^43^ described the role of potassium (K) fertilisation on improving WUE in maize plants. Potentially the slow release of nutrients from wool, including K, may have contributed to the increased WUE in red clover. However, as herbage nutrient status was not explored in this study, further research is required to establish whether this is a potential protective mechanism provided by wool.

### Lucerne plant responses

Lucerne plant height, RWC and specific leaf area were reduced by drought. This, plus increased WUE and root‐to‐shoot ratio associated with droughted plants, indicated that the drought conditions imposed, yielded a water‐stress response. Lower plant height is a recognised response of lucerne to drought.^6^ In contrast, whilst no effect of watering regime on root architecture was observed for lucerne, the root‐to‐shoot ratio was increased by drought. Increasing this ratio is a known drought tolerance strategy utilised by plants,^40^ and may explain the finding reported here. Interestingly, in the current study, droughted lucerne plants grown in the presence of wool never reached < 45% pot capacity (the threshold previously reported to induce water stress in lucerne)^26^ during the 15‐day experimental period. Meanwhile, droughted lucerne grown in compost alone had a pot moisture equivalent to < 45% pot capacity by day 9 of the experimental period. Despite this, there were no significant watering regime‐by‐wool interactions observed for lucerne plant responses. Further study would be required to understand whether the water‐stress threshold to elicit water‐deficit stress would need to be specifically determined for the specific cultivar of lucerne used in the present study, and to understand the interplay between pot moisture and RWC.

Addition of wool to the potting mixture of lucerne plants also reduced RWC and specific leaf area, but also was associated with shorter, less branched root formation with lower surface area. As mentioned earlier, it is possible that increased salinity associated with wool may have contributed to a pseudo‐drought effect, potentially contributing to these changes in lucerne.

The results for lucerne were complex. Under the ANOVA model, drought, but not wool, increased WUE; but addition of wool did reduce lucerne root length, surface area and branching. The regression model identified that lucerne plants with greater root mass had higher WUE, yet there was no significant interaction between root mass and either watering regime, or wool inclusion, on WUE. Deep roots and high root density and wide xylem diameter all can increase water uptake.^42^ Possibly, changes in lucerne root architecture negated any potential benefits from improved nutrition provided by the wool (as theorised for red clover), resulting in no changes in WUE. However, further study is required to understand the effect of wool on lucerne root architecture and WUE.

## CONCLUSION

As we strive to develop methods to maintain food production under increasing extremes of climate change, this study showed that addition of wool to compost delayed the development of low moisture environment in the potting mixture under drought conditions. Furthermore, not only was the establishment of a threshold for water stress delayed, the water was chemically available. The authors believe this is the first instance of using *a*_w_ to explore the water potential of wool used as a soil amendment – specifically addressing whether hygroscopic wool could retain, but also ‘share’ water with plants.

The RWC of red clover was improved by addition of 14% wool, but further benefits of adding wool to the potting media to mitigate plant water status were less clear indicating that there may be additional factors affecting water uptake by plants. Further work is required to understand the dynamics of water availability and plant hydration status, in the presence or absence are wool. Studies relating to the effect of wool used as a soil amendment to grow red clover or lucerne are, to the authors' knowledge, lacking in the current literature, and this work contributes novel findings for these plant species. Future work would need to scale this work up to field‐based trials to confirm its potential agricultural benefits.

## FUNDING INFORMATION

This work was completed as part of the GrOw Wales Green project, led by IBERS, Aberystwyth University in partnership with Hybu Cig Cymru, British Wool, Birch Plastics, Farming Connect, Volac, Wool Testing Authority, and AHDB. The project was funded through the Welsh Government Rural Communities–Rural Development Programme 2014–2020, which is funded by the Welsh Government and the European Union. The analysis of the root imaging data and collation and statistical analysis of results, as part of the manuscript writing process were supported the Joy Welch Scholarship fund 2024–2025, awarded to Siân Bethan MacKintosh.

## CONFLICT OF INTEREST

The authors declare there are no conflicts of interest.

## ETHICS STATEMENT

Not applicable.

## Supporting information


**Figure S1.** The decline in pot moisture for different wool potting media treatments (plant‐free) when stored at 20 °C/12 °C day/night for 32 days. At the start of the experiment, potting media was fully saturated (100% pot capacity). Error bars indicate standard deviation. *P < 0.05, **P < 0.01, ***P < 0.001, NS = not significant.
**Table S1**. Linear regression relationship between potting media moisture and pot capacity, for different wool potting media treatments. The adjusted *R* ^2^ was 96.9% with a standard error of observations of 4.47, and *P* < 0.001. *P > 0.05, **P < 0.01, ***P < 0.001, NS = not significant.
**Figure S2**. The relationship between pot capacity (% of total pot capacity) and potting media moisture content (%) for each of the different wool potting media treatments (no plants). The significant differences between the linear regression slopes are detailed in Supporting Information Table S1.
**Table S2** Multi linear regression analysis of root weight and specific leaf area with water use efficiency (WUE) for red clover and lucerne forage treatments. *P > 0.05, **P < 0.01, ***P < 0.001, NS = not significant.

## Data Availability

The data that support the findings of this study are available from the corresponding author upon reasonable request.
