# Supplementary material for: Grassland forage legumes and drought mitigation – sheep wool application improved soil water availability and enhanced drought–stress responses
Source: J Sci Food Agric. 2026 Jul 8;106(13):8073–84. doi: 10.1002/jsfa.70841 (PMC13543708; doi:10.1002/jsfa.70841)
Supplement: Supplementary file 1 [file JSFA-106-8073-s001.docx]

**Supplementary Data**

**Figure S1**. The decline in pot moisture for different wool potting media treatments (plant-free) when stored at 20 ˚C/12˚C day/night for 32 days. At the start of the experiment, potting media was full saturated (100% pot capacity). Error bars indicate standard deviation. * *P* < 0.05, ** *P* < 0.01, *** *P* < 0.001, NS = non significant.

Plant-Free Potting Mixtures

Pot Moisture (%)

| Days Post-Saturation | 0% Wool | 7% Wool | 14% Wool |
| --- | --- | --- | --- |
| 0 | 59.1333 | 57.4889 | 54.5889 |
| 6 | 46.3778 | 46.0889 | 41.8778 |
| 13 | 35.6222 | 36.3222 | 36.2333 |
| 18 | 30.9778 | 31.8222 | 32.9222 |
| 27 | 20.4 | 20.3444 | 21.8444 |
| 32 | 17.4222 | 23.4 | 23.7556 |

**Table S1**. Linear regression relationship between potting media moisture and pot capacity, for different wool potting media treatments. The adjusted R^2^ was 96.9% with a standard error of observations of 4.47, and P < 0.001. *P >0.05, **P <0.01, ***P <0.001, NS = not significant.

|  | **Estimate** | **S.E.** | **Significance** |
| --- | --- | --- | --- |
| Constant | 1.85 | 1.89 | NS |
| Pot Moisture | 1.6432 | 0.0474 | ******* |
| 7% Wool | -6.88 | 2.93 | ***** |
| 14% Wool | -16.90 | 3.13 | ******* |
| Pot Moisture *10% Wool | 0.2181 | 0.0742 | ****** |
| Pot Moisture *20% Wool | 0.5453 | 0.0816 | ******* |

.

**Figure S2**. The relationship between pot capacity (% of total pot capacity) and potting media moisture content (%) for each of the different wool potting media treatments (no plants). The significant differences between the linear regression slopes are detailed in Supplement Data Table S1.

**Table S2** Multi Linear Regression Analysis of root weight and specific leaf area with water use efficiency (WUE) for red clover and lucerne forage treatments .*P >0.05, **P <0.01, ***P <0.001, NS = not significant.

|  | **Red Clover** | | |  | **Lucerneǂ** | | |
| --- | --- | --- | --- | --- | --- | --- | --- |
|  | **Estimate** | **Adjusted R^2^** | **Significance** |  | **Estimate** | **Adjusted R^2^** | **Significance** |
| **Model** |  |  |  |  |  |  |  |
| *Leaf area + total root wt + watering regime* |  | *48.3%* | ********* |  |  | *58.9%* | ********* |
| Constant | 5.50 |  | ****** |  | 2.220 |  | ** |
| Leaf Area (mm^2^) | -0.00277 |  | NS |  | -0.00168 |  | NS |
| Total root weight (g DM) | 9.28 |  | ****** |  | 5.46 |  | * |
| Watering regime | -2.78 |  | NS |  | -2.093 |  | ***** |
| Leaf Area*watering regime | 0.00259 |  | NS |  | 0.00135 |  | NS |
| Total root weight*watering regime | -6.05 |  | NS |  | 2.53 |  | NS |
|  |  |  |  |  |  |  |  |
| *Leaf area + total root wt + wool content* |  | *19.8%* | NS |  |  | *42.6%* | * |
| Constant | 2.42 |  | NS |  | 0. 76 |  | NS |
| Leaf Area (mm^2^) | -0.00203 |  | NS |  | -0.00076 |  | NS |
| Total root weight (g DM) | 8.63 |  | * |  | 7.41 |  | * |
| Wool content 10% | 3.53 |  | NS |  | 1.49 |  | NS |
| Wool content 20% | 5.97 |  | NS |  | 0. 53 |  | NS |
| Leaf Area*Wool content 10% | 0.00038 |  | NS |  | -0.00233 |  | NS |
| Leaf Area*Wool content 20% | -0.00205 |  | NS |  | -0.00188 |  | NS |
| Total root weight*Wool content 10% | -516 |  | NS |  | 0. 56 |  | NS |
| Total root weight*Wool content 20% | -357 |  | NS |  | 0. 96 |  | NS |

ǂ Two outliers removed.
